# High-Efficiency Ag-Modified ZnO/g-C_3_N_4_ Photocatalyst with 1D-0D-2D Morphology for Methylene Blue Degradation

**DOI:** 10.3390/molecules29102182

**Published:** 2024-05-07

**Authors:** Shuyao Qiu, Jin Li

**Affiliations:** 1 School of Physical Science and Technology, Xinjiang University, Urumqi 830017, China; 107552002160@stu.xju.edu.cn; 2 Xinjiang Key Laboratory of Solid State Physics and Devices, Xinjiang University, Urumqi 830017, China

**Keywords:** photocatalyst, g-C_3_N_4_, ZnO, Ag, methylene blue (MB), Z-type heterojunction

## Abstract

Photocatalysts with different molar ratios of Ag-modified ZnO to g-C_3_N_4_ were prepared through an electrostatic self-assembly method and characterized through techniques such as X-ray diffraction, Fourier transform infrared spectroscopy, and scanning electron microscopy. The resulting Ag-ZnO/g-C_3_N_4_ photocatalysts exhibited a unique 1D-0D-2D morphology and Z-type heterojunction. Moreover, g-C_3_N_4_ nanosheets with large layer spacing were prepared using acid treatment and thermal stripping methods. The Z-type heterostructure and localized surface plasmon resonance effect of Ag nanowires enabled high-speed electron transfer between the materials, while retaining large amounts of active substances, and broadened the light response range. Because of these features, the response current of the materials improved, and their impedance and photoluminescence reduced. Among the synthesized photocatalysts, 0.05Ag-ZnO/g-C_3_N_4_ (molar ratio of g-C_3_N_4_/ZnO: 0.05) exhibited the highest photocatalytic performance under UV–visible light. It degraded 98% of methylene blue in just 30 min, outperforming both g-C_3_N_4_ (21% degradation in 30 min) and Ag-ZnO (84% degradation in 30 min). In addition, 0.05Ag-ZnO/g-C_3_N_4_ demonstrated high cycling stability.

## 1. Introduction

The discharge of organic dyes into water by various industries is a major cause of water pollution and a serious health hazard to society [1,2,3]. A growing awareness of environmental protection has prompted researchers to conduct in-depth studies on green technologies for the photocatalytic degradation of organic dyes [4,5,6]. There are many candidates with excellent performance in the field of photocatalysis, such as SnO_2_ [7], TiO_2_ [8], ZnO [9], etc. Among them, ZnO is a commonly used photocatalyst because of its low cost and environmental friendliness [10,11]. However, the low visible light utilization efficiency, low photogenerated carrier production, and rapid recombination of electron–hole pairs in ZnO limit its photocatalytic performance. These limitations can be overcome by constructing heterojunctions with other materials and doping precious metals (Ag, Cu, Pt, and Pd) into the ZnO structures [12,13,14,15,16,17].

Previous studies have established the novel electronic and photonic properties of two-dimensional layered materials, such as graphene, boron nitride, and graphitic carbon nitride [18,19,20]. Among them, graphitic carbon nitride (g-C_3_N_4_) is an organic polymer semiconductor with a structure similar to graphite, where polyheptazinimide and triazinyl groups form a mesh and the layers are connected by van der Waals forces. It has a bandgap of 2.7 eV and its valence and conduction band positions are at −1.1 eV and +1.6 eV, respectively [21,22,23,24]. Because of its narrow band gap (2.7 eV), excellent thermal stability as well as optical and electrical properties, g-C_3_N_4_ can be used in energy storage, water purification, clean energy preparation, and sensing applications [25,26,27]. However, the rapid recombination of photogenerated electron–hole pairs, small specific surface area, and limited number of active sites due to the stacked lamellar morphology of g-C_3_N_4_ hinder its widescale application. The properties of g-C_3_N_4_ can be modified by fabricating hybrid heterojunction structures with other semiconductors [28,29,30].

Heterojunctions with special morphologies tend to have higher physical and chemical properties and exhibit good performance as photocatalysts, supercapacitors, photodetectors, and other systems. Rajesh et al. synthesized nickel–cobalt-layered double hydroxide (Ni-Co LDH)@Ag nanowire (Ag NWs)/g-C_3_N_4_ nanomaterials using hydrothermal and ultrasonic methods. The addition of Ag nanowires and g-C_3_N_4_ nanosheets accelerated the electron/ion transport in the material, resulting in high electrical conductivity and stability [20]. Sayan Bayan et al. prepared plasmonic hybrid heterojunctions for photodetection [31], in which Ag, ZnO, and g-C_3_N_4_ exhibited nanoparticle, nanorod, and nanosheet morphologies, respectively. Excess electrons were transferred from g-C_3_N_4_ to ZnO, effectively separating the carriers, and effective energy transfer from g-C_3_N_4_ to Ag was realized. Tian et al. [32] synthesized gas-sensitive sensors with Pt nanoparticles/ZnO nanorods/g-C_3_N_4_ lamellae; their gas-sensitive response to ethanol and NO_2_ was enhanced seven-fold and four-fold for the special Pt/ZnO/g-C_3_N_4_ with a special morphology in relation to that for ZnO alone. However, only a few photocatalysts with unique morphologies, such as Ag, ZnO, and g-C_3_N_4_, have been studied. Among them, mpg-C_3_N_4_/Ag/ZnO nanowire/Zn photocatalytic sheets with unique morphologies, prepared by Aydin [33], realized a 94% degradation of Direct Orange 26 in 120 min.

Based on such reported systems, we designed and synthesized Ag-ZnO/g-C_3_N_4_ ternary material with a novel structure. Fluffy g-C_3_N_4_ nanosheets with a large layer spacing were prepared by using the acid-treated precursor method and via thermal stripping. The g-C_3_N_4_ had a zeta potential suitable for complexation with other components. The prepared ternary material has a one-dimensional (1D)–zero-dimensional (0D)–two-dimensional (2D) morphology, in which the ZnO takes the form of 0D nanoparticles, the Ag takes the form of 1D nanowires, and the g-C_3_N_4_ takes the form of 2D nanosheets. The photocatalyst realized the efficient photocatalytic degradation of methylene blue (MB; 98.3% degradation of 20 mg/L MB in 30 min). The synthesized samples were characterized through X-ray diffraction (XRD), X-ray photoelectron spectroscopy (XPS), Fourier transform infrared (FTIR) spectroscopy, scanning electron microscopy (SEM), and zeta potential measurement, whose results demonstrated the successful synthesis of the materials. The synthesized 1D-0D-2D Ag-ZnO/g-C_3_N_4_ photocatalyst enabled the high-speed transfer of electrons between the components. The Ag nanowires act as natural conductors that capture electrons and accelerate electron transfer [34]. The ZnO nanoparticles and fluffy g-C_3_N_4_ nanosheets provided a large number of reactive active sites. Because of the heterojunction structure of the composite material, a high rate of electron–hole separation was achieved, which consequently enhanced the photocatalytic response and reduced the impedance of the photocatalyst. The synthesized Ag-ZnO/g-C_3_N_4_ photocatalyst demonstrated excellent performance toward MB degradation (98.3% degradation within 30 min) and good recycling stability.

## 2. Results and Discussion

### 2.1. Structural, Morphological, and Elemental Characterization

**Figure 1:**
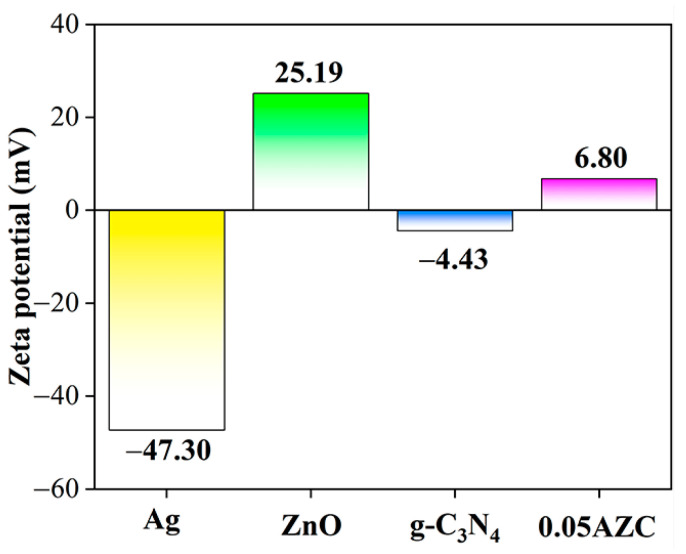
Zeta potential of Ag, ZnO, g-C_3_N_4_, and 0.05AZC.

The zeta potential of each component and 0.05AZC was measured in a neutral environment with a pH of 7 using a Zetasizer Pro (Figure 1). The zeta potentials for Ag, ZnO, and g-C_3_N_4_ were −47.30 mV, +25.19 mV, and −4.43 mV, respectively. Zeta potential reflects the positive and negative charges on the entire surface of a material. In the steady state, materials with the same charge tend to aggregate together, and the corresponding ions in the solution maintain its overall electroneutrality. When subjected to ultrasonic radiation, materials with positive and negative potentials are attracted to each other, leading to their adsorption and subsequent aggregation. In this work, the 0.05AZC potential of the composite material prepared by electrostatic self-assembly was +6.80 mV. This positive potential was attributed to the varying contents of the different components, resulting in an overall positively charged surface for the material. This potential change also confirms the feasibility of the electrostatic self-assembly method and the successful synthesis of composite materials.

**Figure 2:**
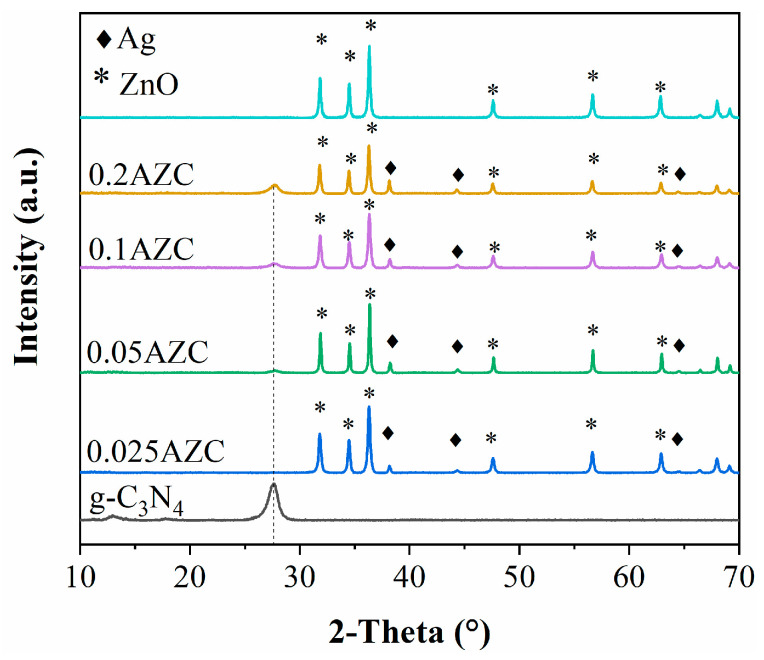
XRD patterns of pure ZnO, AZC, and g-C_3_N_4_.

The XRD patterns of the samples are shown in Figure 2. Three strong characteristic peaks of ZnO are observed at 31.7°, 34.4°, and 36.2°, corresponding to the (100), (002), and (101) crystal faces of the ZnO hexagonal zinc structure, respectively. The characteristic peaks at 47.5°, 56.5°, and 62.8° are associated with the (102), (110), and (103) crystal faces, respectively, which also match well with the standard diffraction patterns of the wurtzite hexagonal structure of ZnO (JCPDS Card No. 99-0011) [35]. The g-C_3_N_4_ has characteristic peaks at 13.16° and 27.7°, which aligns with the standard card (JCPDS Card No. 87-1526) [36]. These peaks reflect the crystal information of the (001) and (002) crystal faces. There were three peaks in the AZC pattern at 38.3°, 44.5°, and 64.5°, which were due to the presence of Ag, that is, the (111), (200), and (220) crystal faces of metallic Ag (JCPDS No. 87-0597) [37]. In all the AZC samples, the diffraction peaks became sharper with increasing g-C_3_N_4_ content, whereas the diffraction peaks of ZnO gradually decreased. None of the synthesized samples exhibited any impurity peaks; the diffraction peaks appeared clear and sharp, indicative of a high degree of crystallinity.

**Figure 3:**
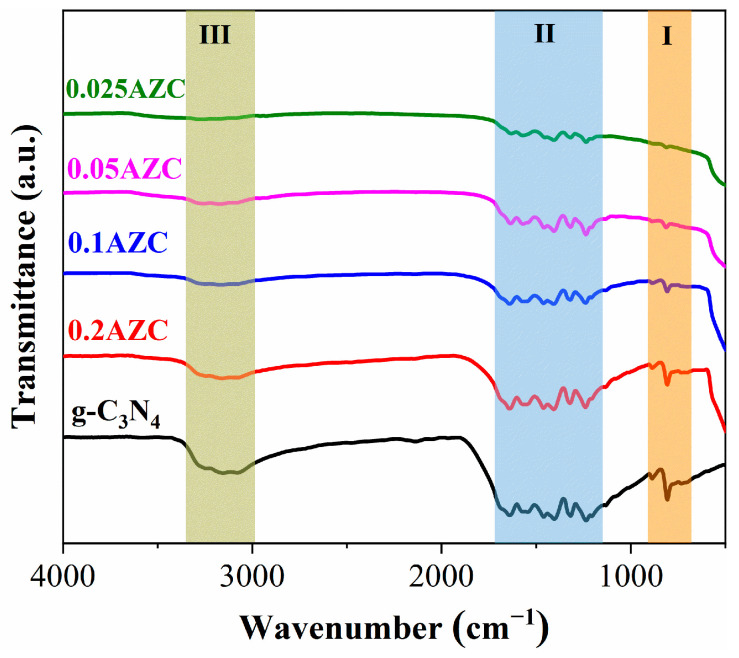
FTIR spectra of AZC and g-C_3_N_4_.

Qualitative and quantitative analyses of the Fourier transform infrared (FTIR) spectra were performed by comparing the positions and intensities of the absorption peaks. g-C_3_N_4_ is an organic polymer semiconductor, and FTIR tests were performed on the samples containing g-C_3_N_4_. The peak near 806 cm^−1^ (I) corresponds to the breathing vibration mode of the tris-triazines (Figure 3); the peak at 886 cm^−1^ corresponds to the bending vibration of the triazine structure, which comprises C and N atoms [38]. The characteristic absorption peaks in the range 1180–1705 cm^−1^ (II) correspond to the variety of vibration modes of the C–N bond in the triazine ring, whereas that at 1568 cm^−1^ corresponds to the telescopic vibration of the sp^2^ orbital C=N double bonds. The characteristic peaks at 1242 cm^−1^, 1324 cm^−1^, 1409 cm^−1^, and 1564 cm^−1^ are caused by the vibration of the C–N bond in the sp^3^ orbital in the aromatic ring structure; the absorption peaks after 3000 cm^−1^ (III) are caused by the O–H and N–H bonds [39,40,41]. The vibrational peak intensity and g-C_3_N_4_ content were observed to be positively correlated. Additionally, at higher content levels of Ag and ZnO in the samples, the characteristic absorption peaks of g-C_3_N_4_ consistently exhibited notable blue shifts. This blue shift phenomenon weakened with increasing g-C_3_N_4_ content.

**Figure 4:**
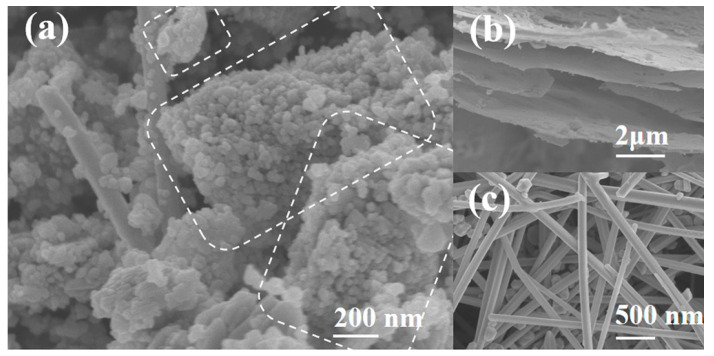
SEM images of (**a**) 0.05AZC; (**b**) g-C_3_N_4_; (**c**) Ag.

The surface morphologies of Ag, ZnO, and 0.05AZC were analyzed using a field-emission scanning electron microscope (SEM), and a clear morphology with depth of field was obtained. The sizes of the samples were measured using a Gatan Digital Micrograph; the diameter of the ZnO nanoparticles was in the range 27–50 nm, with an average diameter of approximately 39.3 nm. The lamellar spacing of the fluffy g-C_3_N_4_ ranged between 844 nm and 1 μm, whereas the average diameter of Ag nanowires was 96.7 nm. In Figure 4a, fluffy g-C_3_N_4_ attached by ZnO is shown inside the white dotted frames. The ZnO particles were uniform in size, with slight agglomeration, and adsorbed between the Ag nanowires and the lamellae of g-C_3_N_4_. Slender Ag nanowires attached to ZnO were interwoven between the sheets of g-C_3_N_4_. The image clearly shows the morphology of the sample, which further confirms the successful synthesis of the composite photocatalysts. The SEM images show good contact between the components, implying that electrons can be transferred between them.

**Figure 5:**
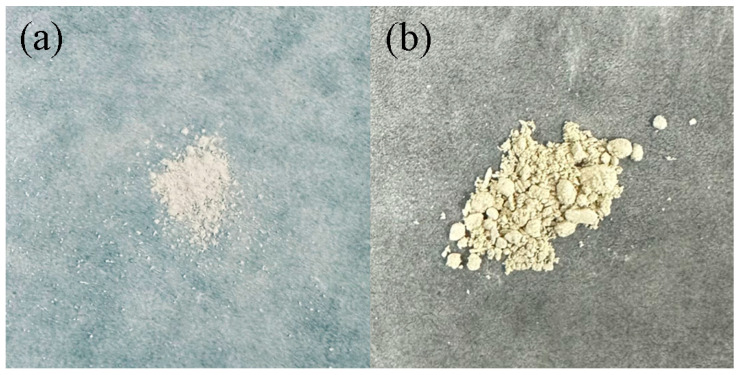
Same weight samples of (**a**) ZnO; (**b**) g-C_3_N_4_.

**Figure 6:**
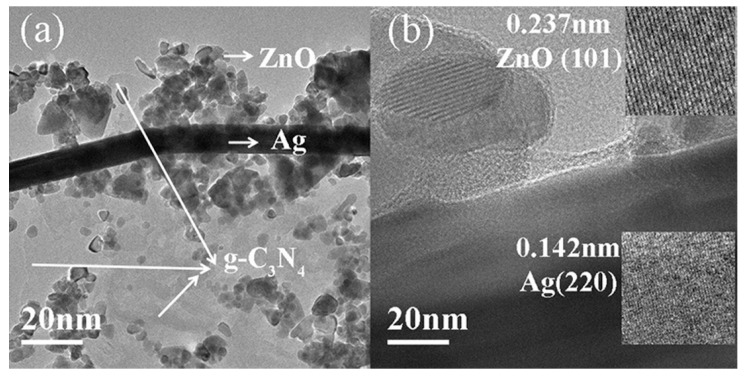
(**a**) TEM and (**b**) HRTEM images of 0.05AZC.

The same weight (0.01 g) samples of ZnO and g-C_3_N_4_ show large volume differences (Figure 5), confirming the fluffiness of g-C_3_N_4_. Transmission electron microscopy (TEM) and high-resolution transmission electron microscopy (HRTEM) images of the samples were obtained to further examine the micromorphology of 0.05AZC (Figure 6). The images revealed that there is good contact between the components. The silver nanowires in Figure 6a have a diameter of approximately 89 nm and the ZnO nanoparticles are 25–50 nm. The fluffy g-C_3_N_4_ appears as an amorphous sheet, which can be better penetrated by high-energy electron beams. Consequently, it exhibits a lighter color in the TEM images. The crystal structure of the samples is also reflected in the HRTEM image (Figure 6b), where clear lattice streaks are observed for Ag and ZnO, with the lattice fringes with a spacing of 0.237 nm corresponding to the (101) crystal faces of ZnO, and the lattice fringes with a spacing of 0.142 nm corresponding to the (220) crystal faces of Ag.

**Figure 7:**
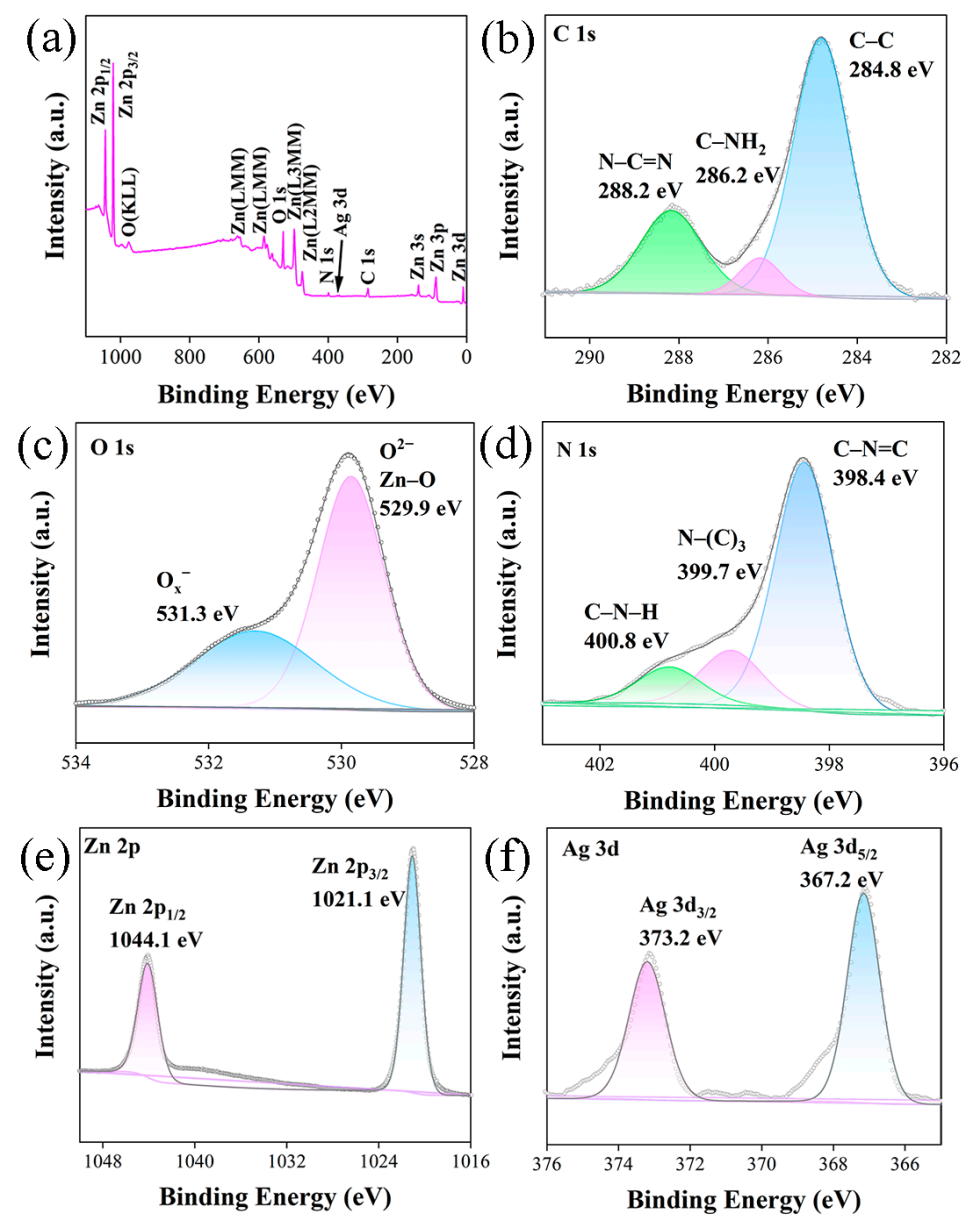
XPS of 0.05AZC: (**a**) survey; (**b**) C 1s;s (**c**) O 1; (**d**) N 1s; (**e**) Zn 2p; (**f**) Ag 3d.

XPS is a common tool for analyzing the chemical elemental composition of materials and their surface valence states. The XPS survey spectrum of the 0.05AZC sample (Figure 7a) provides orbital information of the elements contained in the sample. The full spectrum contains peaks of C, N, O, Zn, and Ag; there are no other stray peaks. The C 1s energy spectrum shows three peaks at binding energies of 288.20, 286.20, and 284.80 eV (Figure 7b), which correspond to C–C/C=C, C–NH_2_, and N–C=N in the aromatic carbon–nitride heterocyclic ring. This indicates the successful synthesis of g-C_3_N_4_. The energy spectrum of O 1s (Figure 7c) exhibits two characteristic peaks observed at binding energies of 531.30 and 529.85 eV. The characteristic peak at 529.85 eV originates from ZnO, whereas the characteristic peak at 531.30 eV is related to C–O and –OH in H_2_O [42]. Additionally, the peak corresponding to O^2−^ indicates hydroxyl adsorption induced by structural defects, which is important for photocatalysis. The energy spectrum of N 1s (Figure 7d) has characteristic peaks at binding energies of 400.80, 399.70, and 398.40 eV, which correspond to the amino group (C–NH_x_) in the heptazine skeleton [43], the three-coordinated N–(C)_3_, and the bi-coordinated C–N=C with sp^2^ hybridization of the N atoms in the aromatic ring, respectively [44]. The energy spectrum of Zn 2p (Figure 7e) has two characteristic peaks at binding energies of 1044.10 eV and 1021.10 eV, corresponding to Zn 2p_1/2_ and Zn 2p_3/2_, respectively. In the XPS spectrum of Ag 3 d (Figure 7f), Ag 3d_3/2_ is positioned at 373.20 eV, while Ag 3d_5/2_ is located at 367.20 eV. These peaks correspond to the metal Ag [45,46]. There was poor fitting to the left of the two characteristic peaks, probably due to the Ag–O formed by a small amount of Ag oxidation. XPS reflects the surface valence state information of the elements. From the above analysis, we confirmed the successful synthesis of heterojunctions.

**Figure 8:**
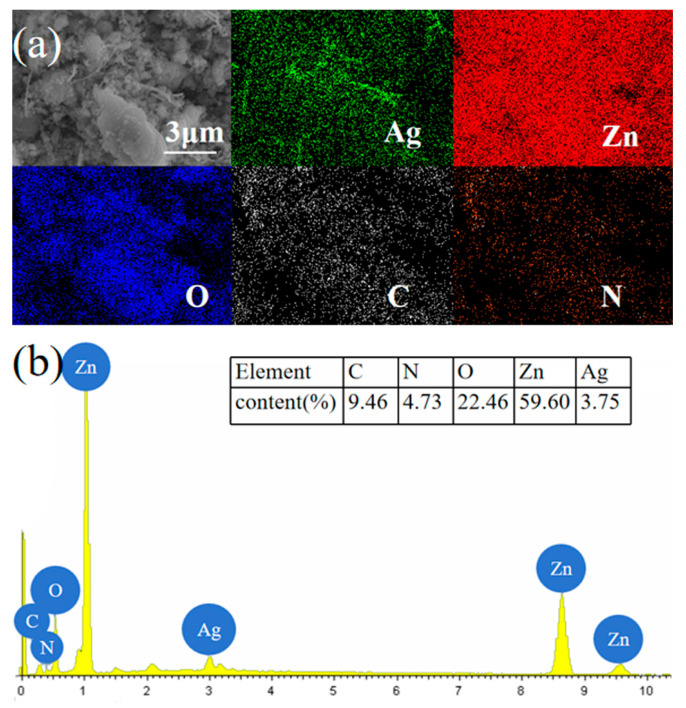
Mapping (**a**) and EDS (**b**) spectra of 0.05AZC.

Energy-dispersive spectroscopy (EDS) was utilized to determine the proportion of each element in the material. Mapping was used to analyze the distribution and enrichment of each element in the material. After selecting the region of interest in the microscope, the material is excited, causing the electrons of the atoms to jump, subsequently emitting characteristic X-rays. By analyzing these X-rays, the photomultiplier and amplifier convert the optical and electrical signals. The intensity of the electron beam on the fluorescent screen is then controlled, showing the mapping scanning image and EDS results. In general, a stronger fluorescence signal of the element in the mapping image indicates that the element is enriched in that particular area. Zn and O were enriched within the region from the mapping (Figure 8a) because of the high amount of ZnO in the material. Elemental Ag was enriched in the form of nanowires, and elemental C and N were present in smaller amounts, showing sheet-like enrichment. These results agree with those of the FESEM analysis. EDS results are often related to the selected region of interest. Therefore, when preparing the materials, the components should be evenly dispersed. Moreover, while selecting an area of interest, well-dispersed areas should be targeted. The proportion of each component in the samples was calculated using EDS analysis. The EDS spectrum (Figure 8b) of the photocatalyst reflects the presence and content of Zn (59.60 wt.%), O (22.46 wt.%), C (9.46 wt.%), Ag (3.75 wt.%), and N (4.73 wt.%) elements in the 0.05AZC sample.

### 2.2. Optical and Electrochemical Properties

**Figure 9:**
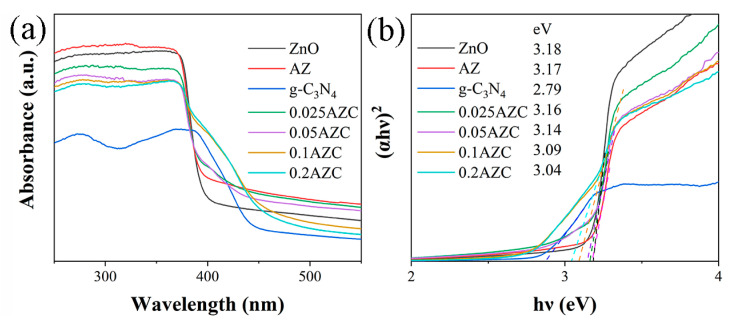
(**a**) UV–Vis DRS and (**b**) Tacu of ZnO, g-C_3_N_4_, AZ, and AZC.

Visible light ranges from approximately 380 to 800 nm [47,48]. The photoelectric properties of the samples were determined using a UV–Vis spectrophotometer in the wavelength range 250–750 nm. When the energy required for the excitation of electrons is small, a redshift phenomenon occurs, and the peak intensity of the UV–Vis spectrum decreases accordingly. Figure 9a shows the UV–Vis diffuse reflectance spectra (DRS) of the material. In contrast to that of ZnO, the UV–Vis absorption intensity of AZ was enhanced by the addition of Ag in the range of less than 330 nm and longer than 400 nm; this enhancement could be attributed to the localized surface plasmon resonance (LSPR) effect of the noble metals [49]. Compared to ZnO, g-C_3_N_4_ shows a lower intensity in the range 250–380 nm and a higher intensity in the range 380–430 nm, with a strong redshift, which implies that g-C_3_N_4_ has better visible light absorption. Compared with AZ, the AZC samples exhibited different degrees of absorption enhancement in the range 380–435 nm; the UV–Vis absorption of AZC shifted to a longer wavelength (redshift). According to eV = 1240/λ, g-C_3_N_4_ (2.79 eV) absorbs and utilizes light ≤ 440 nm, while 0.05AZC (3.14 eV) absorbs and utilizes light ≤ 395 nm. For the AZC samples, the photogenerated electrons produced by g-C_3_N_4_ after absorbing visible light could be transferred through the heterojunction to ZnO and Ag. Therefore, the addition of g-C_3_N_4_ improved the visible light utilization of the material to some extent.

Using the UV–Vis absorption data and Equation (1), Tacu diagrams of ZnO, g-C_3_N_4_, AZ, and AZC were constructed.

(1) $${\left(\alpha hv\right)}^{2}=A\times (hv-E_{g})$$

where *α* is the absorption coefficient, h is Planck’s constant, v is the frequency of light, $E_{g}$ is the optical band gap, and A is the constant. Figure 9b shows that the band gap of the prepared g-C_3_N_4_ is approximately 2.79 eV and that for ZnO is approximately 3.18 eV. With the addition of g-C_3_N_4_ and Ag, the bandgap of the prepared AZC gradually decreases. The band gap of 0.05AZC is 3.14 eV. This confirms the successful preparation of the ternary samples and its possession of a suitable band gap.

**Figure 10:**
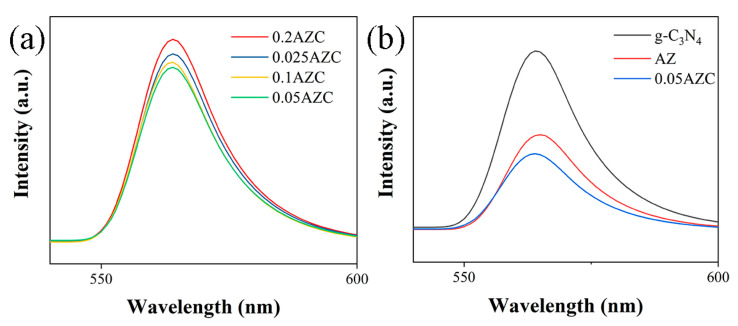
Photoluminescence emission spectra of (**a**) AZC; (**b**) g-C_3_N_4_, AZ, and 0.05AZC.

The photoluminescence (PL) of the samples was measured at an excitation wavelength of 290 nm; the PL emission spectra are shown in Figure 10. Figure 10a shows the photoluminescence of different samples of AZC, with 0.05AZC exhibiting the lowest fluorescence intensity. The photoluminescence of g-C_3_N_4_ was strong (Figure 10b). g-C_3_N_4_ possesses a narrow bandgap, making it easily excited and capable of generating a large number of photogenerated electrons and holes. When the photogenerated electrons jump to the valence band, they compound the photogenerated holes, resulting in the release of numerous photons. Consequently, g-C_3_N_4_ with a narrow band gap can produce a large number of photogenerated carriers, leading to a high photogenerated electron–hole composite ratio [50]. However, after recombining the Ag-modified ZnO with g-C_3_N_4_, the photoluminescence intensity of the composite decreases. This is attributed to the unique band structure, wherein electrons can move rapidly in the heterojunction. This movement extends the life of the photogenerated carriers and reduces the recombination rate of the light-generated electron–hole pairs.

**Figure 11:**
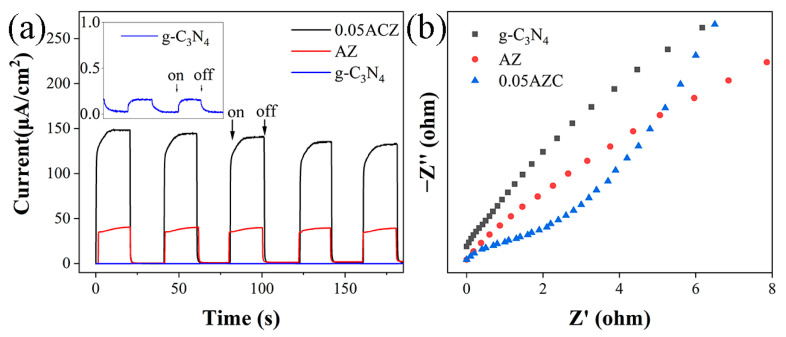
(**a**) Transient photocurrent responses and (**b**) EIS of g-C_3_N_4_, AZ, and 0.05AZC.

The transient photocurrent test reflects the response of catalysts to light and can be used to evaluate the conductivity and carrier mobility of the materials. When light irradiates the surface of the materials, the electrons in the valence band of the materials are excited to the conduction band. These electrons then move directionally under the influence of the electric field generating a current, that is, a light-generated current. In this test, the photocurrent response test was conducted at 20 s intervals; the results are shown in Figure 11a. Among them, the photocurrent response of g-C_3_N_4_ was poor; 0.05AZC exhibited good response speed and strength. When the 300 W xenon lamp radiated the electrolytic tank, a significant change in current was observed. Blocking the light source resulted in the return of the current signal to a smooth state. After the recombination with g-C_3_N_4_, the photocurrent response of 0.05AZC significantly increased. This enhancement can be attributed to the heterostructure of the composite materials, which enabled the photogenerated electrons to move rapidly on the band, extending the life of the photogenerated carriers and generating large photocurrents. This result was consistent with the PL test results.

The samples were tested for impedance, which is an important indicator of the electrical conductivity of the materials. The smaller the arc radius in electrochemical impedance spectroscopy (EIS), the smaller the impedance of the materials [51]. The test results of the samples are shown in Figure 11b. The impedance curve of the 0.05AZC sample has an arc of minimum radius, which indicates that the formation of ternary samples successfully improves the electrochemical properties of the 0.05AZC. This is of considerable significance to the photocatalytic performance [52].

### 2.3. Photocatalytic Properties

**Figure 12:**
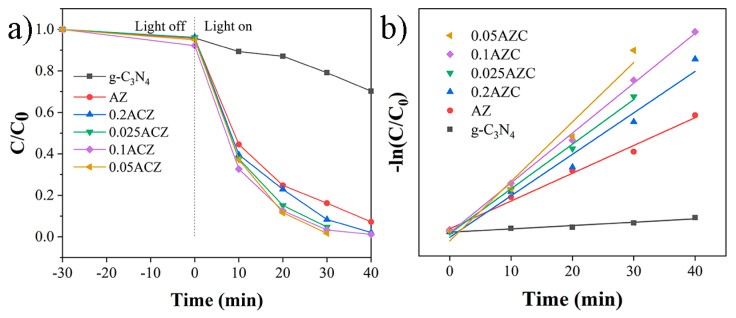
(**a**) Photocatalytic degradation of MB and (**b**) first-order kinetic plots of g-C_3_N_4_, AZ, and AZC.

The degree of the photocatalytic degradation of MB is shown in Figure 12a. Each material is observed to have different degrees of adsorption to MB during the dark reaction. After turning on the xenon lamp, 5 mL of the liquid was collected every 10 min. The UV–Vis absorption intensity of the liquid was determined using an ultraviolet spectrophotometer. The degree of photocatalytic degradation was calculated using Equation (2) [53]. Figure 12a shows the photocatalytic degradation process. The first-order kinetic curve was calculated using Equation (3) [54] (Figure 12b).

(2) $$D(\%)=(1-C/C_{0})\times 100\%$$

(3) $$-In(C/C_{0})=Kt$$

The pollutant concentration without degradation was recorded as *C*_0_, and the concentration after light exposure (min) was recorded as *C*, where *K* is a constant and is the slope of first-order dynamics. In Figure 12a, 0.05AZC exhibited the best photocatalytic degradation of 98.3% of MB at 30 min. In contrast, g-C_3_N_4_ was 21% degraded at 30 min, probably because of its structure and narrow band gap, making the photoelectron–hole pairs unable to efficiently degrade MB. The degradation of AZ was 84% after 30 min and increased to 98.3% with the addition of g-C_3_N_4_. The curves of each sample fit well (Figure 12b) and the process of pollutant degradation complied with the first-order kinetics of degradation. The slope (*K*) of g-C_3_N_4_, AZ, 0.2AZC, 0.025AZC, 0.1AZC, and 0.05AZC are 0.00743, 0.06181, 0.0923, 0.09932, 0.11094, and 0.13231 min^−1^, respectively. The kinetic fitting curve of 0.05AZC has a maximum slope of 0.13231 min^−1^. By comparing these trends, we found that the addition of g-C_3_N_4_ improved the degree and speed of photocatalytic degradation of MB.

**Figure 13:**
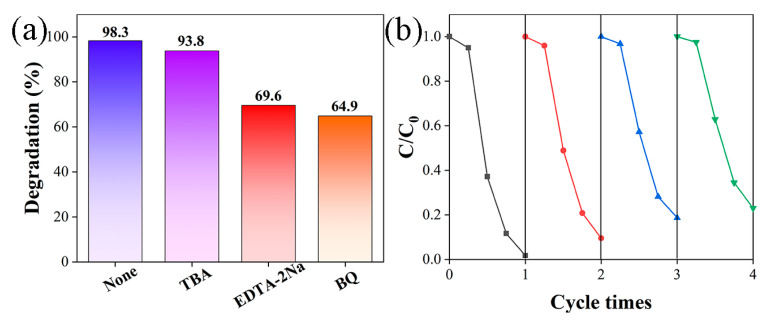
(**a**) Free radical capture experiment and (**b**) photocatalytic degradation cycle of 0.05AZC.

Capture experiments are widely used in photocatalytic experiments, where the photocatalytic efficiency deteriorates when the main functional free radicals are trapped. This has significant implications for determining the types of radicals produced by a photocatalyst. TBA, EDTA-2Na, and BQ were selected as hydroxyl radical (·OH), photogenerated hole (h^+^), and superoxide radical (·O^2−^), respectively. The results of the capture experiment (Figure 13b) indicate that the degradation degree of 0.05AZC is 98.3%. When TBA, EDTA-2 Na, and BQ were added, the degradation rate was 93.8%, 69.6%, and 64.9%, respectively. This revealed that the holes (h^+^) and superoxide radicals (·O^2−^) played the main degradation role in the ternary samples; the contribution of ·OH is small. The photocatalytic cycle of the 0.05AZC sample, which exhibited the best performance, was also tested. The degradation efficiency of the sample decreased with an increasing number of cycles (Figure 13a); however, overall, the 0.05AZC sample was stable.

Compared with other photocatalytic performance studies on the ternary systems of Ag, ZnO, and g-C_3_N_4_ (Table 1), the ternary samples 0.05 AZC prepared in this work showed better photocatalytic performance. We believe that this may be related to the morphology of the samples prepared in this study. The specific photocatalytic mechanism is elaborated in a later section.

### 2.4. Photocatalytic Mechanism

The positions of the valence and conduction bands of the materials were calculated by combining the absolute electronegativities and the bandgap widths of the materials calculated in the Tacu diagram (Figure 9b) according to Equations (4) and (5):

(4) $$E_{VB}=\chi -E_{e}+0.5E_{g}$$

(5) $$E_{CB}=E_{VB}-E_{g}$$

where χ is the absolute electronegativity of the semiconductor. The χ and the bandgap ($E_{g}$) of ZnO are 5.79 eV [58] and 3.18 eV, respectively. The χ and $E_{g}$ of g-C_3_N_4_ are 4.73 eV [59] and 2.79 eV, respectively. $E_{e}$ is a constant that is the energy of a free electron on the hydrogen scale (approximately 4.5 eV). The calculated $E_{VB}$ for ZnO is 2.88 eV and the $E_{CB}$ is −0.3 eV. The $E_{VB}$ for g-C_3_N_4_ is 1.63 eV and the $E_{CB}$ is −1.16 eV, which is in agreement with what is reported in the literature [60]. The standard redox potential E_0_(O_2_/·O_2_^−^) = −0.33 eV/vs. NHE, E_0_(OH^−^/·OH)=1.99 eV/vs. NHE. ·O_2_^−^ can be generated from O_2_ only when the $E_{CB}$ of the material is more negative than E_0_(O_2_/·O_2_^−^). ·OH can be generated from OH^−^ when $E_{VB}$ is more positive than E_0_(OH^−^/·OH). In the heterojunction constructed in this work, the $E_{CB}$ of ZnO is not sufficient to produce ·O_2_^−^, whereas the $E_{VB}$ of g-C_3_N_4_ is not sufficient to produce ·OH. According to the free radical trapping experiments, the main active substances in the 0.05AZC photocatalyst are h^+^ and ·O_2_^−^, and the secondary active substances are ·OH. Therefore, 0.05AZC has a Z-type heterojunction structure, where e^−^ in the conduction band of ZnO combines with h^+^ in the valence band of g-C_3_N_4_. The e^−^ and h^+^, which are in the conduction band of g-C_3_N_4_ and the valence band of ZnO, are continuously enriched.

**Scheme 1:**
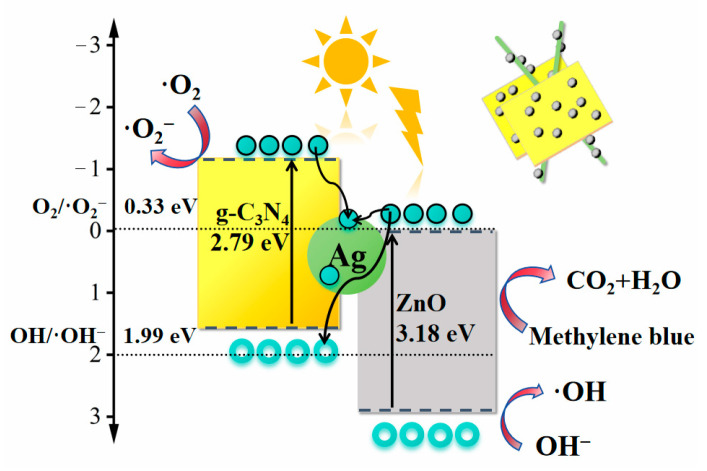
Photocatalytic degradation mechanism over 0.05AZC under sunlight irradiation.

The photocatalytic mechanism is shown in Scheme 1. g-C_3_N_4_ with a narrow band gap forms a type-Z heterojunction after contact. The fluffy g-C_3_N_4_ increases the number of active reaction sites, and the special structure of the semiconductor heterojunction and Ag nanowire improves the electron transfer speed of the material. Meanwhile, the LSPR effect of Ag [49] increases the UV–visible light absorption in a certain wavelength range. g-C_3_N_4_ also increases the visible light absorption. Ternary materials have sufficient active sites, good light absorption and utilization abilities, and strong oxidation of active substances to efficiently degrade MB. When light is irradiated on composite materials, the light energy simultaneously stimulates the electrons in the ZnO and g-C_3_N_4_ semiconductors, exciting them from the valence band to the conduction band, while creating holes in the valence band of the semiconductor. The e^−^ in the conduction band of ZnO combines with h^+^ in the valence band of g-C_3_N_4_. In addition, h^+^ in the valence band of ZnO can oxidize H_2_O or –OH into ·OH, which can oxidize organic pollutants to CO_2_ and H_2_O. Metallic Ag nanowires are natural metal wires, and Ag atoms can serve as electron traps to capture photogenerated electrons, enabling electrons to move between materials and improving the electron–hole separation rate. e^−^ in the conduction band of g-C_3_N_4_ can also react with O_2_ adsorbed on the surface of ZnO/g-C_3_N_4_ to form ·O_2_^−^ and H_2_O_2_. The h^+^, ·O_2_^−^, and ·OH species are strongly oxidizing, and can directly oxidize organic pollutants.

**Scheme 2:**
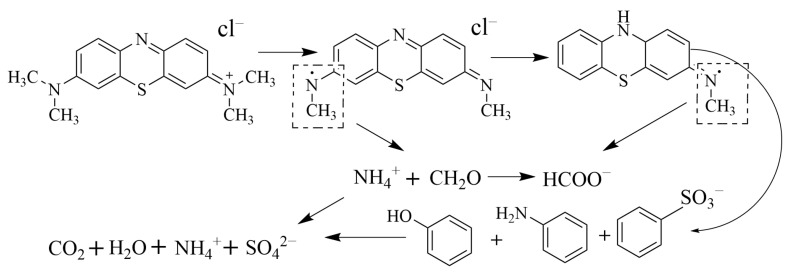
Main process of photocatalytic decomposition of MB by 0.05 AZC.

Scheme 2 visualizes the products of the photocatalytic degradation process of MB: MB is oxidized by the free radical active substances in the material, removing a methyl group at both ends of the structure and forming a lone electron around N; the double bonds absorb energy and become single bonds. The aromatic ring gradually decomposes to generate NH_4_^+^ and HCHO, which are oxidized to HCOO^−^, and HCOO^−^ can further form carboxylates; phenol, benzenesulfonate, and other substances are oxidized, and ultimately converted into harmless products of CO_2_, H_2_O, and ions.

## 3. Materials and Methods

### 3.1. Materials

Zinc acetate (Zn(Ac)_2_·2H_2_O), sodium hydroxide (NaOH), silver nitrate (AgNO_3_), ethylene glycol (CH_2_OH), sodium sulfide nonahydrate (Na_2_S·9H_2_O), polyvinyl pyrrolidone (PVP), acetone (C_3_H_6_O), melamine (C_3_H_6_N_6_), phosphorous acid (H_3_PO_3_), and ethylene glycol (EG) were procured from Macklin company. All reagents were of analytical grade and used as is without purification.

### 3.2. Synthesis of 0D ZnO Nanoparticles

ZnO nanoparticles were synthesized using a simple hydrothermal method. First, 0.5 M NaOH solution was dropped into 0.5 M Zn(AC)_2_·2H_2_O solution at a rate of 30 mL/h, followed by continuous stirring for 30 min to ensure homogeneous mixing. After centrifugation of the mixed solution, a white precipitate was obtained. The precipitate was washed repeatedly with ethanol and deionized (DI) water, followed by drying at 80 °C for 2 h in an oven. The precipitate was calcined at 250 °C for 3 h in a muffle furnace at a heating rate of 5 °C/min.

### 3.3. Synthesis of 1D Ag Nanowires

A solvothermal method was used to obtain 1D Ag nanowires in the experiment. Initially, quantitated AgNO_3_ was dissolved in (CH_2_OH)_2_ to prepare a 0.1 M solution. Na_2_S·9H_2_O (30 mM) and PVP (0.15 M) were used as corrosion inhibitors and dispersants to protect the metal and regulate grain crystal growth. They were then dissolved in an appropriate amount of (CH_2_OH)_2_ and homogeneously dispersed via ultrasonication. The mixed solution was then injected into the AgNO_3_ solution at a rate of 45 mL/h. The obtained mixed solution was transferred to a reactor and placed in a high-temperature and high-pressure environment at 155 °C for 1.5 h. The suspension was centrifuged, and the precipitate was washed with C_3_H_6_O, dispersed in C_2_H_5_OH, and stored at 4 °C.

### 3.4. Synthesis of 2D g-C_3_N_4_ Nanosheets

First, 1 g of C_3_H_6_N_6_ and 1.2 g of H_3_PO_3_ were sequentially placed in a beaker with 100 mL of deionized (DI) water. A water bath was used to maintain 80 °C of the environment to ensure the dissolution of drugs, with continuous stirring for 1 h. Subsequently, the above solution was poured into the polytetrafluoroethylene reaction liner and placed into a high-temperature and high-pressure reactor for 10 h in a vacuum oven at 180 °C. The suspension was centrifuged and washed several times with DI water and C_2_H_5_OH, and the precipitate was dried to obtain a white, self-assembled precursor. The precursor was evenly spread inside the magnetic boat, and after the lid was put on, the magnetic boat was wrapped with 0.1 mm carbon paper to prevent excessive loss of samples during the calcination process. The magnetic boat was placed inside a tube furnace and N_2_ was introduced as a protective atmosphere, and heated to 550 °C for 4 h at a temperature increase rate of 5 °C/min; the abovementioned calcination process was repeated once. Finally, a fluffy yellow powder of g-C_3_N_4_ was obtained.

### 3.5. Synthesis of Photocatalyst

An electrostatic self-assembly method was utilized to prepare the 1D-0D-2D photocatalyst. The Ag content was fixed at 8 wt.%. Samples with g-C_3_N_4_:ZnO molar ratios of 0.025, 0.05, 0.1, and 0.2 were dispersed in ethanol, sonicated for 30 min, and stirred for 30 min. The precipitate was centrifuged and dried (denoted as 0.025AZC, 0.05AZC, 0.1AZC, and 0.2AZC, respectively, where A, Z, and C represent Ag, ZnO, and g-C_3_N_4_, respectively). For comparison, a binary Ag-ZnO photocatalyst without g-C_3_N_4_ was prepared and labeled as AZ.

### 3.6. Characterizations

A zeta potential meter (Zetasizer Pro, Malvern Panalytical, London, UK) was used to analyze the surface charge of samples. To obtain the phase structure information, XRD (Rigaku/smartLab Bruker D8 Advance, Saarland, Germany) was conducted. The apparent morphology of all samples was determined through SEM (ZEISS sigma550, Oberkochen, Germany). TEM (Hitachi HT7800, Tokyo, Japan) was performed to analyze the micromorphology, diffraction images, and lattice stripe spacing of each component of the composites. To verify the chemical composition of the materials, XPS (Thermo ESCALAB Xi+, Waltham, MA, USA) was performed. EDS (Hitachi Quantax70, Tokyo, Japan) was conducted to analyze the contents of elements in the materials. Furthermore, a UV–Vis spectrophotometer (Hitachi U-3010, Tokyo, Japan) was used to perform liquid and solid UV evaluations of the prepared samples. Photoluminescence (PL; FLS1000/FS5, Edinburgh, UK) analysis was performed to study the illumination characteristics of the samples. In addition, the transient photocurrent test was carried out on an electrochemical workstation equipped with a 300 W xenon lamp (Perfectlight, Beijing, China). Photocatalytic experiments were also conducted using a xenon lamp as the light source.

### 3.7. Photocatalytic Degradation Test

In this study, MB was used as the target pollutant for photocatalytic degradation. A total volume of 20 mg/L MB solution was prepared, and 10 mg samples was dispersed in 50 mL of the MB solution. Under continuous magnetic stirring, the dark reaction was allowed to proceed for 30 min to achieve the adsorption–desorption equilibrium. After the dark reaction, 5 mL of the liquid was used for testing. A Xe lamp (PLS-SXE300/300UV) was used as the light source, and the solution was irradiated and stirred. A total volume of 5 mL of liquid was collected every 10 min until the solution changed from dark blue to colorless. The MB concentrations in the test solutions were determined using a UV–visible spectrophotometer (Hitachi U-3010, Tokyo, Japan).

## 4. Conclusions

In this study, Ag-modified ZnO/g-C_3_N_4_ composites were prepared via electrostatic self-assembly, wherein Ag nanowires were synthesized by the solvothermal method, ZnO by the hydrothermal method, and g-C_3_N_4_ nanosheets by acid treatment and secondary calcination thermal stripping. XRD, FTIR spectroscopy, EDS, UV–Vis spectrophotometry, and an electrochemical workstation were performed to comprehensively analyze the structure, morphology, photoelectric properties, and photocatalytic properties of the samples. The results confirmed that the ternary material with the 1D-0D-2D morphology has good contact among the components, forming a Z-type semiconductor heterojunction with a band gap of ∼3.14 eV. The 0.05AZC sample exhibited the highest photocatalytic performance, with a degradation of ∼98.3% of 20 mg/L MB in 30 min. The radical trapping experiment results revealed that the main active substances in the photocatalyst are superoxide radicals (·O_2_^−^) and holes (h^+^). Based on the UV–Vis absorption spectroscopy results, a possible photocatalytic mechanism for 0.05AZC was proposed.

## Figures and Tables

**Table 1:** Comparison of photodegradation of methylene blue between some current studies.

Sr. No.	Catalyst	Morphology	Heterojunction	C (mg/L)	Light Source	T (min)	Degradation (%)	Ref.
1	Ag-ZnO/g-C_3_N_4_	0D-2D	II	10	250 W Hg lamp	60	100	[55]
2	Ag/ZnO/S-g-C_3_N_4_	0D-2D	II	10	Sunlight (57–63 Klux)	60	98	[56]
3	7Ag-ZnO/S-g-C_3_N_4_	0D-2D	II	10	Sunlight (57–63 Klux)	40	97	[57]
4	0.05Ag-ZnO/g-C_3_N_4_	1D-0D-2D	Z	20	300 W xenon lamp	30	98.3	This work

## Data Availability

The data presented in this study are available on request from the corresponding author.

## References

[1] Alguacil F.J., López F.A. (2021). Organic Dyes versus Adsorption Processing. Molecules.

[2] Zafar Z., Fatima R., Kim J.-O. (2021). Experimental studies on water matrix and influence of textile effluents on photocatalytic degradation of organic wastewater using Fe–TiO_2_ nanotubes: Towards commercial application. Environ. Res..

[3] Choi M., Singh N., Son S., Kim J.H., Kang M., Park S.H., Choi D.H., Hong C.S., Kim J.S. (2023). A post-synthetically modified porous organic polymer for photocatalytic water purification. Mater. Chem. Front..

[4] Garazhian Z., Rezaeifard A., Jafarpour M., Farrokhi A. (2020). {Mo_72_Fe_30_} Nanoclusters for the Visible-Light-Driven Photocatalytic Degradation of Organic Dyes. ACS Appl. Nano Mater..

[5] Li X., Chen X., Li C., Xu Z., Jiang B. (2023). Emulsifier-modulated covalent organic frameworks for photocatalytic degradation of organic dyes. New J. Chem..

[6] Zhou Y., Elchalakani M., Liu H., Briseghella B., Sun C. (2022). Photocatalytic concrete for degrading organic dyes in water. Environ. Sci. Pollut. Res..

[7] Shao T., Cao X., Dong J., Ning J., Zhang F., Wang X., Cheng Y., Kou H., Zhang W. (2022). Study on the Photocatalytic Properties of Flower-Shaped SnO_2_. Nanomaterials.

[8] Jeon J., Kweon D.H., Jang B.J., Ju M.J., Baek J. (2020). Enhancing the Photocatalytic Activity of TiO_2_ Catalysts. Adv. Sustain. Syst..

[9] Cao C., Zhang B., Lin S. (2022). p-type ZnO for photocatalytic water splitting. APL Mater..

[10] Kumaran N.N., Muraleedharan K. (2017). Photocatalytic activity of ZnO and Sr^2+^ doped ZnO nanoparticles. J. Water Process Eng..

[11] Suresh S., Subash B., Karthikeyan S. (2017). Electrical, optical and photocatalytic properties of Ti-loaded ZnO/ZnO and Ti-loaded ZnO nanospheres. J. Iran. Chem. Society.

[12] Mendoza-Mendoza E., Nuñez-Briones A.G., García-Cerda L.A., Peralta-Rodríguez R.D., Montes-Luna A.J. (2018). One-step synthesis of ZnO and Ag/ZnO heterostructures and their photocatalytic activity. Ceram. Int..

[13] Li H., Ding J., Cai S., Zhang W., Zhang X., Wu T., Wang C., Foss M., Yang R. (2022). Plasmon-enhanced photocatalytic properties of Au/ZnO nanowires. Appl. Surf. Sci..

[14] Arifin M., Roza L., Fauzia V. (2019). Bayberry-like Pt nanoparticle decorated ZnO nanorods for the photocatalytic application. Results Phys..

[15] Lee S.J., Jung H.J., Koutavarapu R., Lee S.H., Arumugam M., Kim J.H., Choi M.Y. (2019). ZnO supported Au/Pd bimetallic nanocomposites for plasmon improved photocatalytic activity for methylene blue degradation under visible light irradiation. Appl. Surf. Sci..

[16] Peng J., Lu T., Ming H., Ding Z., Yu Z., Zhang J., Hou Y. (2019). Enhanced Photocatalytic Ozonation of Phenol by Ag/ZnO Nanocomposites. Catalysts.

[17] Sarma B., Sarma B.K. (2017). Fabrication of Ag/ZnO heterostructure and the role of surface coverage of ZnO microrods by Ag nanoparticles on the photophysical and photocatalytic properties of the metal-semiconductor system. Appl. Surf. Sci..

[18] Guo Y., Liu C., Yin Q., Wei C., Lin S., Hoffman T.B., Zhao Y., Edgar J.H., Chen Q., Lau S.P. (2016). Distinctive in-Plane Cleavage Behaviors of Two-Dimensional Layered Materials. ACS Nano.

[19] Liu L., An W., Gu F., Cui L., He X., Fan M. (2023). 2D layered materials: Structures, synthesis, and electrocatalytic applications. Green Chem..

[20] Rajesh D., Francis M.K., Bhargav P.B., Nafis A., Balaji C. (2022). 2D layered nickel-cobalt double hydroxide nano sheets @ 1D silver nanowire-graphitic carbon nitrides for high performance super capacitors. J. Alloys Compd..

[21] Naseri A., Samadi M., Pourjavadi A., Moshfegh A.Z., Ramakrishna S. (2017). Graphitic carbon nitride (g-C_3_N_4_)-based photocatalysts for solar hydrogen generation: Recent advances and future development directions. J. Mater. Chem. A.

[22] Zhou L., Zhang Z., Li M., Wang Q., Gao J., Li K., Lei L. (2021). Graphitic carbon nitride (g-C_3_N_4_) as a sustainable heterogeneous photocatalyst for metal free and oxygen-tolerant photo-atom transfer radical polymerization (photo-ATRP). Green Chem..

[23] Mamba G., Mishra A.K. (2016). Graphitic carbon nitride (g-C_3_N_4_) nanocomposites: A new and exciting generation of visible light driven photocatalysts for environmental pollution remediation. Appl. Catal. B Environ..

[24] Li H., Yin S., Sato T., Wang Y. (2016). Enhanced Photocatalytic Performance of Luminescent g-C_3_N_4_ Photocatalyst in Darkroom. Nanoscale Res. Lett..

[25] Sano T., Koike K., Hori T., Hirakawa T., Ohko Y., Takeuchi K. (2016). Removal of methyl mercaptan with highly-mobile silver on graphitic carbon-nitride (g-C_3_N_4_) photocatalyst. Appl. Catal. B Environ..

[26] Jiang X., Qiao K., Feng Y., Sun L., Jiang N., Wang J. (2022). Self-assembled synthesis of porous sulfur-doped g-C_3_N_4_ nanotubes with efficient photocatalytic degradation activity for tetracycline. J. Photochem. Photobiol. A Chem..

[27] Xu J., Chen Y., Chen M., Wang J., Wang L. (2022). In situ growth strategy synthesis of single-atom nickel/sulfur co-doped g-C_3_N_4_ for efficient photocatalytic tetracycline degradation and CO_2_ reduction. Chem. Eng. J..

[28] Li W., Zhou L., Xie L., Kang K., Xu J., Chai X. (2022). N-Fe-Gd co-doped TiO_2_/g-C_3_N_4_ nanosheet hybrid composites with superior photocatalytic dye degradation. Adv. Compos. Hybrid Mater..

[29] Wang J., Wang G., Wang X., Wu Y., Su Y., Tang H. (2019). 3D/2D direct Z-scheme heterojunctions of hierarchical TiO_2_ microflowers/g-C_3_N_4_ nanosheets with enhanced charge carrier separation for photocatalytic H_2_ evolution. Carbon.

[30] Wang L., Yang T., Peng L., Zhang Q., She X., Tang H., Liu Q. (2022). Dual transfer channels of photo-carriers in 2D/2D/2D sandwich-like ZnIn_2_S_4_/g-C_3_N_4_/Ti_3_C_2_ MXene S-scheme/Schottky heterojunction for boosting photocatalytic H_2_ evolution. Chin. J. Catal..

[31] Bayan S., Gogurla N., Ghorai A., Ray S.K. (2019). Förster Resonance Energy Transfer Mediated Charge Separation in Plasmonic 2D/1D Hybrid Heterojunctions of Ag–C_3_N_4_/ZnO for Enhanced Photodetection. ACS Appl. Nano Mater..

[32] Tian H., Fan H., Ma J., Liu Z., Ma L., Lei S., Fang J., Long C. (2018). Pt-decorated zinc oxide nanorod arrays with graphitic carbon nitride nanosheets for highly efficient dual-functional gas sensing. J. Hazard. Mater..

[33] Hassani A., Faraji M., Eghbali P. (2020). Facile fabrication of mpg-C_3_N_4_/Ag/ZnO nanowires/Zn photocatalyst plates for photodegradation of dye pollutant. J. Photochem. Photobiol. A Chem..

[34] Orcutt E.K., Varapragasam S.J., Peterson Z.C., Andriolo J.M., Skinner J.L., Grumstrup E.M. (2023). Ultrafast Charge Injection in Silver-Modified Graphitic Carbon Nitride. ACS Appl. Mater. Interfaces.

[35] Chen D., Huang S., Huang R., Zhang Q., Le T.-T., Cheng E., Yue R., Hu Z., Chen Z. (2019). Construction of Ni-doped SnO_2_-SnS_2_ heterojunctions with synergistic effect for enhanced photodegradation activity. J. Hazard. Mater..

[36] Paul D.R., Sharma R., Nehra S.P., Sharma A. (2019). Effect of calcination temperature, pH and catalyst loading on photodegradation efficiency of urea derived graphitic carbon nitride towards methylene blue dye solution. RSC Adv..

[37] da Silva R.R., Yang M., Choi S.-I., Chi M., Luo M., Zhang C., Li Z.-Y., Camargo P.H.C., Ribeiro S.J.L., Xia Y. (2016). Facile Synthesis of Sub-20 nm Silver Nanowires through a Bromide-Mediated Polyol Method. ACS Nano.

[38] Sun Z., Fischer J.M.T.A., Li Q., Hu J., Tang Q., Wang H., Wu Z., Hankel M., Searles D.J., Wang L. (2017). Enhanced CO_2_ photocatalytic reduction on alkali-decorated graphitic carbon nitride. Appl. Catal. B Environ..

[39] Guo S., Deng Z., Li M., Jiang B., Tian C., Pan Q., Fu H. (2016). Phosphorus-Doped Carbon Nitride Tubes with a Layered Micro-nanostructure for Enhanced Visible-Light Photocatalytic Hydrogen Evolution. Angew. Chem. Int. Ed..

[40] Guo W., Zhang J., Li G., Xu C. (2019). Enhanced photocatalytic activity of P-type (K, Fe) co-doped g-C_3_N_4_ synthesized in self-generated NH_3_ atmosphere. Appl. Surf. Sci..

[41] Petit C., Seredych M., Bandosz T.J. (2009). Revisiting the chemistry of graphite oxides and its effect on ammonia adsorption. J. Mater. Chem..

[42] Zhao S., Zheng M., Sun H., Li S., Pan Q., Guo Y. (2020). Construction of heterostructured g-C_3_N_4_/ZnO/cellulose and its antibacterial activity: Experimental and theoretical investigations. Dalton Trans..

[43] Zhang J., Zhang M., Zhang G., Wang X. (2012). Synthesis of Carbon Nitride Semiconductors in Sulfur Flux for Water Photoredox Catalysis. ACS Catal..

[44] Qin H., Zuo Y., Jin J., Wang W., Xu Y., Cui L., Dang H. (2019). ZnO nanorod arrays grown on g-C_3_N_4_ micro-sheets for enhanced visible light photocatalytic H_2_ evolution. RSC Adv..

[45] Abdel Messih M.F., Ahmed M.A., Soltan A., Anis S.S. (2019). Synthesis and characterization of novel Ag/ZnO nanoparticles for photocatalytic degradation of methylene blue under UV and solar irradiation. J. Phys. Chem. Solids.

[46] Yao X., Zhang W., Huang J., Du Z., Hong X., Chen X., Hu X., Wang X. (2020). Enhanced photocatalytic nitrogen fixation of Ag/B-doped g-C_3_N_4_ nanosheets by one-step in-situ decomposition-thermal polymerization method. Appl. Catal. A Gen..

[47] Qi S., Fei L., Zuo R., Wang Y., Wu Y. (2014). Graphene nanocluster decorated niobium oxide nanofibers for visible light photocatalytic applications. J. Mater. Chem. A.

[48] Bozhynov V., Kovacs Z., Cisar P., Urban J. (2021). Application of Visible Aquaphotomics for the Evaluation of Dissolved Chemical Concentrations in Aqueous Solutions. Photonics.

[49] Khademalrasool M., Farbod M., Talebzadeh M.D. (2021). Investigation of shape effect of silver nanostructures and governing physical mechanisms on photo-activity: Zinc oxide/silver plasmonic photocatalyst. Adv. Powder Technol..

[50] Gao J., Zhou Y., Li Z., Yan S., Wang N., Zou Z. (2012). High-yield synthesis of millimetre-long, semiconducting carbon nitride nanotubes with intense photoluminescence emission and reproducible photoconductivity. Nanoscale.

[51] Wang Y., Ding K., Xu R., Yu D., Wang W., Gao P., Liu B. (2020). Fabrication of BiVO_4_/BiPO_4_/GO composite photocatalytic material for the visible light-driven degradation. J. Clean. Prod..

[52] Wen J., Xie J., Yang Z., Shen R., Li H., Luo X., Chen X., Li X. (2017). Fabricating the Robust g-C_3_N_4_ Nanosheets/Carbons/NiS Multiple Heterojunctions for Enhanced Photocatalytic H_2_ Generation: An Insight into the Trifunctional Roles of Nanocarbons. ACS Sustain. Chem. Eng..

[53] Luo W., Ying J., Yu S., Yang X., Jia Y., Chen M., Zhang H., Gao J., Li Y., Mai Y.-W. (2020). ZnS:Cu powders with strong visible-light photocatalysis and pyro-catalysis for room-temperature dye decomposition. Ceram. Int..

[54] Segovia M., Alegría M., Aliaga J., Celedon S., Ballesteros L., Sotomayor-Torres C., González G., Benavente E. (2019). Heterostructured 2D ZnO hybrid nanocomposites sensitized with cubic Cu_2_O nanoparticles for sunlight photocatalysis. J. Mater. Sci..

[55] Nguyen T.T.A., Dao T.C.V., Vu A.-T. (2024). Controlling the physical properties of Ag/ZnO/g-C_3_N_4_ nanocomposite by the calcination procedure for enhancing the photocatalytic efficiency. Ceram. Int..

[56] Iqbal S., Ahmad N., Javed M., Qamar M.A., Bahadur A., Ali S., Ahmad Z., Irfan R.M., Liu G., Akbar M.B. (2021). Designing highly potential photocatalytic comprising silver deposited ZnO NPs with sulfurized graphitic carbon nitride (Ag/ZnO/S-g-C_3_N_4_) ternary composite. J. Environ. Chem. Eng..

[57] Iqbal S., Bahadur A., Javed M., Hakami O., Irfan R.M., Ahmad Z., AlObaid A., Al-Anazy M.M., Baghdadi H.B., Abd-Rabboh H.S.M. (2021). Design Ag-doped ZnO heterostructure photocatalyst with sulfurized graphitic C_3_N_4_ showing enhanced photocatalytic activity. Mater. Sci. Eng. B.

[58] Xu Y., Schoonen M.A.A. (2000). The absolute energy positions of conduction and valence bands of selected semiconducting minerals. Am. Mineral..

[59] Shang Y., Chen X., Liu W., Tan P., Chen H., Wu L., Ma C., Xiong X., Pan J. (2017). Photocorrosion inhibition and high-efficiency photoactivity of porous g-C_3_N_4_/Ag_2_CrO_4_ composites by simple microemulsion-assisted co-precipitation method. Appl. Catal. B Environ..

[60] Duan S.-F., Tao C.-L., Geng Y.-Y., Yao X.-Q., Kang X.-W., Su J.-Z., Rodríguez-Gutiérrez I., Kan M., Romero M., Sun Y. (2019). Phosphorus-doped Isotype g-C_3_N_4_/g-C_3_N_4_: An Efficient Charge Transfer System for Photoelectrochemical Water Oxidation. ChemCatChem.

